# Comparison the effect of Sleep Positioning on Cardiorespiratory Rate in Noninvasive Ventilated Premature Infants

**DOI:** 10.5812/nms.10318

**Published:** 2013-06-27

**Authors:** Fatemeh Ghorbani, Maliheh Asadollahi, Sousan Valizadeh

**Affiliations:** 1 Department of Pediatric Nursing, Tabriz University of Medical Sciences, Tabriz, IR Iran

**Keywords:** Premature infant, Heart rate, Respiratory rate, Prone position, Supine position

## Abstract

**Background:**

Results of several studies suggest that prone position is beneficial in improving the preterm infants’ cardio-respiratory status. Previous studies showed opposite results, and also there is not any available clear study about the effect of this position on cardio-respiratory rates of Nasal Continuous Positive Airway Pressure (N-CPAP) treating premature infants.

**Objectives:**

This study aimed at comparing supine and prone positions on cardio-respiratory rates of premature infants with respiratory distress syndrome (RDS) who were treated using N-CPAP.

**Patients and Methods:**

This was a cross over study which was performed in 2010 on 44 hospitalized 29-34 weeks gestation premature infants who were receiving N-CPAP in Neonatal Intensive Care Unit of Al-Zahra Hospital of Tabriz University of Medical Sciences. Infants were randomly assigned into two groups, and the first group was placed in prone at first and then in supine, and the position of second group was at first supine and then prone. Infants’ Heart Rate (HR) and Respiratory Rate (RR) were assessed three times in each position for 30 minutes. The data was recorded in a data-collection form, and demographic data was analyzed using t test, Chi square and Fisher exact test. Also, repeated measurement ANOVA and Tukey post-hoc tests were used.

**Results:**

There was a significant difference in HR and RR of premature infants who were similar in gestational age and clinical condition and placed in two positions. Premature infants’ HR and RR became lower at prone position than supine in both groups. So it can be concluded that prone position could decrease infants HR and RR, but supine position might increase them (P < 0.05).

**Conclusion:**

Our findings support prone positioning for premature infants. Therefore, it is advisable to NICU staff that if there is no obstacle for changing the infant’s position, prone position in infants with respiratory complications during receiving N-CPAP in NICU can be useful. Regarding the fact that prone position is a risk factor for sudden infant death syndrome , prone position should be only used when the newborn is being supervised carefully.

## 1. Background

Soon after a premature infant is born, it’s a big challenge to maintain previous functional patterns against different stimuli (1). Today, prematurity is the most important cause of admission in Neonatal Intensive Care Units (NICUs). Prematurity of lung tissue and respiratory distress syndrome are the common problems in premature infants which illustrate the need for special attentions for the respiratory cares (2). Cardio-respiratory interaction has been considered an important indicator of development in infants, and it has also been reported that heart rate fluctuations may exist at the respiratory frequencies even in the absence of respiration (3). Also positioning of preterm infants is a basic neonatal nursing care that includes supine, prone, side-lying, and head up tilted position. Several studies have reported a variety of outcomes affected by different body positioning of preterm infants. Prone positioning was shown to have many advantages for prematurely born infants. Preterm infants in the prone position spend less time awake and more time quiet and asleep. It is reported that prone position can improve lungs and cardio-respiratory development, organize digestive functions and facilitate improvement of respiratory status. Most of caregivers believe that premature and low birth weight infants are at ease and sleep comfortably in prone position too (4). However there are various results about the effect of sleeping position on premature infants’ cardio-respiratory status. Some studies found that in prone position the infants’ cardio-respiratory status is improved, while some other studies showed that supine position had better effect. In some studies there was no significant difference among different positions too. For example Maynard and Leipal showed that premature infants’ cardio-respiratory rates were lower in prone than supine position (5); however, Fifer and Ammaria found opposite results in their studies. They showed that infants’ cardio-respiratory rates increases more in prone than in supine position (6, 7). Also in the Levy’s study there was no significant difference in infants’ heart rate (HR) and respiration rate (RR) when they were placed in two different positions (8). Although studies shows advantages and disadvantages for both the supine and prone positions among premature infants to their physiological outcomes, there is a trend remaining toward keeping premature infants in a supine position due to increased risk of sudden infant death syndrome in infants placed in a prone position, and it is said that prematurely born infants compared with those born at term have a higher incidence of sudden infant death syndrome (SIDS). So since 1992 the American Academy of Pediatrics (AAP) has recommended that infants, including premature infants who do not have respiratory distress and are being readied to be discharged from hospital, be placed to sleep in a nonprone position to reduce the risk of SIDS (4). Regarding the fact that physiological mechanisms underlie SIDS are still unknown, and AAP’s advice about condition of infants who are avoided to be placed prone, it can be concluded that avoiding prone position is necessary for infants who do not have any respiratory distress, and are specially taken care at home. Therefore it is clear that placing premature infants with respiratory complications in prone position is safe when they are being monitored and supervised carefully in NICUs.

## 2. Objectives

Considering opposite results of previous studies and since no clear study has been found about the effect of premature infants’ sleeping position on their cardio-respiratory parameters as they are under Nasal Continuous Positive Airway Pressure (N-CPAP) treatment, this study was designed to compare the effect of prone and supine positions on cardio-respiratory rate of premature infants who were treated using N-CPAP.

## 3. Patients and Methods

This interventional study was performed in cross over method on premature infants who were hospitalized in NICU of Al-Zahra teaching hospital of Tabriz University of Medical Sciences from May to November 2010. Inclusion criteria were 29-34 weeks gestational age, having respiratory distress syndrome, being under N-CPAP but without severe respiratory distress syndrome, no need for intubation, being NPO, and lack of congenital heart disease, or any physical defect, which is a contraindication to changing the position. Exclusion criteria were the need for intubation, pneumothorax, and need for chest tube insertion. This single-blind study was performed by an experienced nurse of NICU. The nurse was not blinded to treatment and groups; while, infants’ parents knew nothing about classifying the groups. To collect the data we used a two-section data-collection form. The first part, 12 questions were about infants’ demographic information, and infant’s HR and in the second part RR were recorded three times every 10 minutes in each position. The infants were randomly assigned into two groups. The first qualified infant was put in the first group by coin toss-porn and was placed in prone as the first position, then every other infant was placed in the other group alternately as they were enrolled. To eliminate any effect of changing the position, the data was collected after stabilization period of 15 minutes (as wash out time), i.e after 15 minutes infants’ HR and RR were recorded in the data collection form 3 times in the first position every 10 minutes in 30 minutes totally. After 30 minutes infants’ position was changed in both groups. i.e in the first group which the first position was prone, the position was changed to supine, and in the second group which the first position was supine, it was changed to prone. After changing the position, researcher waited 15 minutes again, and then recorded the data in new position, so every infant was placed 30 minutes in each position and 15 minutes in wash out period for every position (120 minutes totally). To record HR, the pulse oximeter – Masimo set – was used. The pulse oximeter sets were calibrated once a year by an expert and during collecting data they were calibrated once more. Infants’ RR was counted in one minute by the researcher through observation of their chest and abdomen movements. To ensure the reliability of infants’ RR, counting the breaths of 10 infants was performed simultaneously by another coworker in NICU. The calculated correlation coefficient was acceptable (r = 95%).

According to the Kohen scale on sample size of interventional studies, regarding U = 1, α = 0.05, Effect size = 50% and power of test = 99%, 22 infants for each group were selected (9). (U = number of groups - 1).

### 3.1. Ethical considerations

This work was approved by the Medical Research Ethics Committee of Tabriz University of Medical Sciences. Informed consents were obtained from the parents of eligible infants at first, and data collection was performed after receiving permission of the hospital and unit authorities as well.

### 3.2. Date analysis

The data was collected in the specified forms and was analyzed using SPSS software Version 13 (SPSS, Chicago, IL, USA). Demographic data such as birth weight, body temperature, gestational age, and Apgar score were reported as mean (SD) and compared in two groups using t test. Gender, and frequency (percentage) of delivery was compared in two groups by Chi square and Fisher exact tests. For comparing difference in infant’s HR and RR between the two groups during the three different assessment times we used repeated measurement ANOVA, followed by Tukey post-hoc testing. The test validity was considered statistically significant at P < 0.05 level.

## 4. Results

**Diagram 1.**
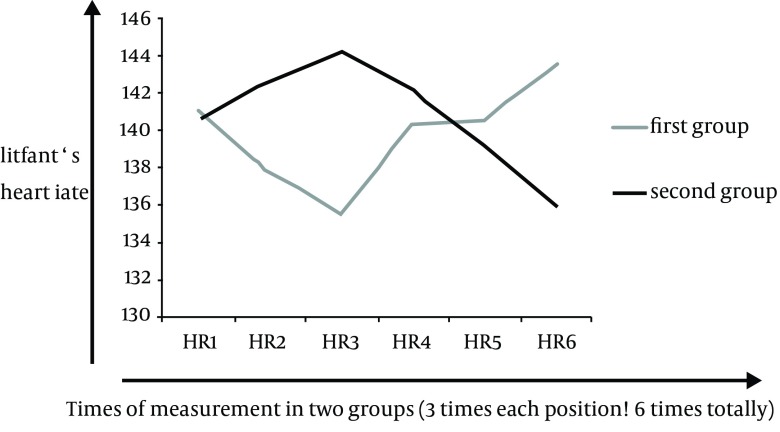
Infants’ HR in Two Groups When Their Position Was Changed from Prone to Supine and Vice Versa

**Diagram 2.**
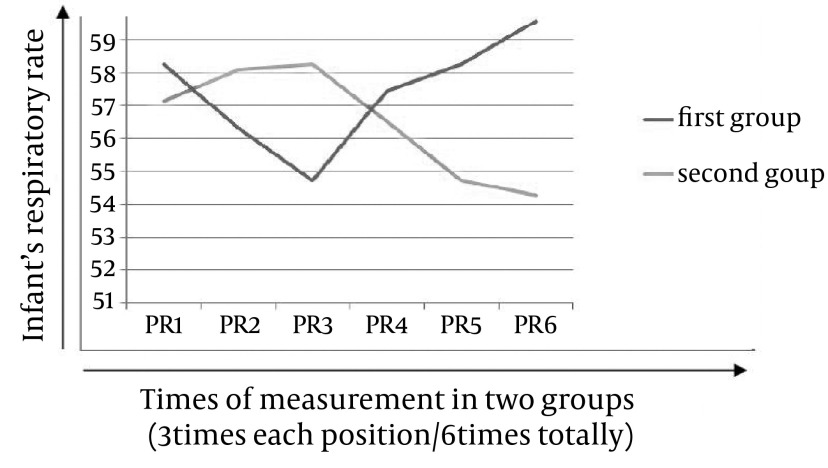
Infants’ RR in Two Groups When Their Position Was Changed from Prone to Supine, and Vice Versa

Demographic information such as infant’s birth weight, body temperature and gestational age in two groups has been summarized in Table 1. Mean and standard deviation of infants’ HR were different in both groups, i.e. during three times of measuring, these values were descending in prone, and ascending in supine positions in both of groups (Table 2).Mean and standard deviation of infants’ RR was different in both groups too, i.e. during three times of measuring; these values were descending in prone and ascending in supine position (Table 3). Comparing infants’ HR and RR in both groups suggested that the results of repeated measurement test were statistically significant (P < 0.05) i.e. in both of the two groups infants’ HR (Diagram 1) and RR (Diagram 2) decreased when their position was changed from supine to prone.

**Table 1.** Comparison of Demographic Characteristics of Infant’s in Two Groups

Variable	Group 1 (n = 22)	CI^a^95%		Group 2 (n = 22)	CI 95%		P Value
		lower	higher		lower	higher		
**Birth Weight g, (Mean ± SD)**	1688 ± 5 13.7	1460.83	1916.44	1665 ± 416.9	1480.56	1850.32	0.07
**Gestational age, week, (Mean ± SD)**	31.45 ± 1.43	30.82	32.09	31.23 ± 1.37	30.62	31.84	0.59
**Gender, No. (%)**							0.10
Female	10 (45.5%)			6 (27.3%)			
Male	12 (54.5%)			16 (72.7%)			
**Type of delivery, No. (%)**							0.50
C/S	14 (63.6%)			15 (68.2%)			
NVD	8 (36.4%)		7 (31.8%)				
**Apgar score**							
**First minute**	7.14 ± 2	6.23	8.05	7.1 ± 2.11	6.25	8.12	0.95
**Fifth minute**	8.86 ± 1.16	8.42	9.4	8.71 ± 1.52	8.10	9.44	0.41
**Body temperature, Mean ± SD**	36.83 ± 0.13	36.77	36.89	36.86 ± 0.12	36.81	36.92	0.08

^a^ Confidence Interval

**Table 2.** Comparison of Means and Standard Deviations of Infants’ HR in Two Groups

	Infants’ HR, Mean ±SD			Statistical tests results	
	First time	Second time	Third time	F	P Value
**First group**				F_ (1,42)_ = 37.04	0.002
First position (prone)	141.09 ± 16.12	138.23 ± 15.35	135 ± 15.04		
Second position (supine)	140.64 ± 14.40	142.73 ± 15.35	144.27 ± 13.09		
**Second group**				F_ (1,42)_ = 38.69	0.001
First position (supine)	140.63 ± 17.12	140.59 ± 16.22	143.55 ± 15.00		
Second position (prone)	141.08 ± 16.12	138.33 ± 15.34	135.55 ± 15.39		

**Table 3.** Comparison of Means and Standard Deviations of Infants’ RR in Two Groups

	Infants’ RR			Statistical tests results	
	First time	Second time	Third time	F	P Value
**First group**				F_(1,42)_= 37.05	0.002
First position (prone)	58.72 ± 14.07	56.23 ± 14.95	54.37 ± 14.03		
Second position (supine)	57.41 ± 13.53	58.09 ± 14.11	58.72 ± 13.99		
**Second group**				F(_1,42)_= 29.78	0.008
First position (supine)	57.54 ± 15.06	58.72 ± 14.13	59.55 ± 14.28		
Second position (prone)	58.41 ± 14.07	56.32 ± 14.59	54.73 ± 14.29		

## 5. Discussion

Our study showed that the mean value of HR and RR in premature infants with respiratory distress syndrome who were treated using N-CPAP decreased in prone position, therefore their tachypnea and tachycardia reduced and infants became more stable in prone rather than supine position. Prone position has been considered as the therapeutic maneuver in the past three decades. Some of authors believe that this position improves oxygenation and reduces energy expenditure in premature infants. Although association of the prone position with SIDS as one of the risk factors of SIDS, causes some concerns about it, and it is recommended to avoid the prone position by AAP but results of some studies have shown that prone position has many advantages for infants when they are cured in NICUs and are monitored by an expert staff in NICU to protect them from SIDS. Maynard et al. (10) studied 10 premature infants with the gestational age of 26 weeks. Seven of infants did not need ventilation and three of them were under CPAP. They concluded that there was a significant difference between infants’ HR when they were placed in prone or supine position. In prone position infants’ HR was significantly lower than supine. Leipala et al. (5) reported that among 20 premature infants with the gestational age of 29 weeks, there was a significant difference between the mean values of RR in prone or supine position. In their study six participants were dependent on oxygen and 14 were independent. Balaguer et al (11) in a systematic review about the effect of positioning on amount of Saturation of Peripheral Oxygen (SPO2), showed that in several studies SPO2 was increased in the prone position 1.18 to 4.36% during the intervention (prone position). Also Yao et al. (12) showed that preterm infants 1 and 6 hours after weaning from mechanical ventilation had a higher PaO2 in prone position compared to supine position. On the contrary Fifer et al. (7) compared prone and supine positions in 20 premature infants who were fed PO and did not take complementary oxygen. The results of their study suggested that infants’ HR in prone was significantly higher than that in supine position. It seems that the observed difference in results is due to differences in participants’ clinical condition i.e. in Fifer’s study, infants were fed completely, and data was collected after feeding, while in our study all of participants were NPO and were depended on oxygen. Findings of Ammaria et al. (6) showed that in 32 premature and LBW infants who did not need complementary oxygen, cardio-respiratory rate was significantly higher in prone than supine position. Levy et al. (8) studied infants who were fed every 3 hours, did not need any complementary oxygen, and were ready to get discharged. They did not report any significant difference in infants’ respiratory function in prone and supine positions. Curely et al. (13) showed that prone positioning does not significantly improve clinical outcomes in pediatric patients with lung injuries too. By comparing the results of present and other studies (5, 10, 11, 14) we can conclude that advantages of prone position in infants who need complementary oxygen are more noticeable. Most of studies also reported that prone position is useful in infants with respiratory problems which may be due to the decreasing pressure of abdominal organs on diaphragm in prone position letting it move freely.

In the present study we found that when premature infants with respiratory complications and similar clinical conditions were placed in prone as first or second position, their respiratory and heart rates reduced, so their tachypnea and tachycardia improved and they got more stable and calm. According to the findings, we can conclude that prone position as a simple, noninvasive and free of charge method can be helpful in stabilizing premature infants’ status with respiratory distress syndrome. Therefore regarding the results of the present study, the nurses of NICU could be advised to put the premature infants under N-CPAP in prone position while they are being monitored continually and there is no contraindication for changing the infant’s position. We should bear in mind that although placing infants in the prone position may improve cardio-respiratory function, but the association of SIDS with prone position must always be considered, and infants should only be placed in this position when they are under continuous cardio-respiratory and O2 saturation monitoring. The present study was performed on 29-34 weeks gestational age infants, therefore the results may not be generalized to all premature infants. Considering the limitations of this study, it is useful to perform more studies in more extended gestational age range. Also it is possible to investigate the effect of body position on feeding tolerance during N-CPAP treatment as well.
